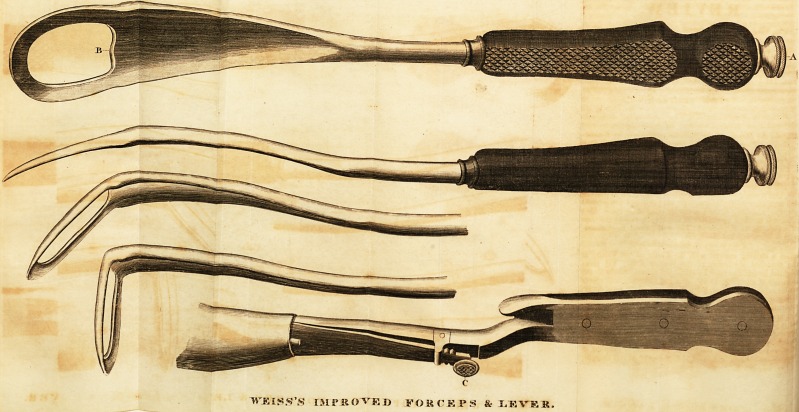# Intelligence, Correspondence, &c.

**Published:** 1825-07-01

**Authors:** 


### XVI.
### INTELLIGENCE, CORRESPONDENCE, &c.

To Dr. Gibson we return many thanks for his interesting communication, and also for his friendly letter, dated "Camp near Severndroog," in the East Indies. His communication will be properly disposed of—and we wish him health till that long-anticipated period, when—

——————— toil and danger o'er,
He'll anchor on his native shore.

Mr. Jowett will see in the present number what he wishes.

*Medical Science in South America.*

There is every reason to believe that, now when the leaden yoke of Old Spain is thrown off, there will be a rapid diffusion of the arts, sciences, and literature of Europe, and more especially of England, throughout those vast regions that stretch from the Isthmus of Darien to the dreary shores of Patagonia.—Our countrymen are already dispersing themselves in all directions on both sides of the Andes, and they will carry with them and make known, through the humbler periodicals of the Southern Continent of America, much of what circulates in the numerous journals of their native land. We had little idea that our humble labours, in the medical department of human knowledge, were already known on the banks of La Plata, when the following letter was presented to the Editor of this Journal, by the hands of our Vice-Consul, C. Griffiths, Esq. from the President of the Academy of Medicine and Natural Science of Buenos Ayres. The Editor feels peculiar gratification in receiving this spontaneous token of approbation from an Academy not yet hackneyed in the ways of similar institutions in the old world, and begs, on behalf of his associates and himself, to acknowledge their grateful sense of the honour conferred, as well as of the flattering expressions accompanying it.

"Señor,

"La Academia de Medicina y Ciencias naturales de Buenos Ayres, deseando procurarse la amistad y correspondencia de los sabios, determinò presentar à V. el diploma de Socio de esta institucion en sesion de 7 de Diciembre del año 1822, que en esta ocasion se remite. Coneste tributo espontaneo pagado al merito elevado que distingue la persona de V., y à a la grande reputacion que le hàn ganado sus interesantes trabajos en las ciencias medicas, la Academia ha querido reconocer tambien que esta parte del Mundo nuevo estimaba debidamente al digno autor de la *Revista medico-quirurgica, y del tratado sobre las enfermedacles de los climas tropicales.* En consecuencia de estos sentimentos es que el Presidènte de la dicha Corporacion, obrando conforme à su acuerdo, dirije à V. el diploma adjunto, y espera que serà aceptado como prulba del alto respeto que le profesa.

"Tengo el honor de firmarme con toda consideracion. de V.

Señor,

Su muy atento humilde servidor,

MANUEL MORENO,

*To the Editor of the Medico-Chirurgical Review.* Presidte. de Acada."

*Invalids going to the Continent for the Winter.* A physician who has resided several years in one of the healthiest and most beautiful cities of Italy, could accommodate an invalid in his own house (which is large, airy, and finely situated) and attend to his health, if required.—Further particulars may be learnt by letter, *post paid,* to Dr. James Johnson.

*Associated Apothecaries and Surgeon Apothecaries of England and Wales.* The Annual General Meeting of this Association will be holden at the Crown and Anchor Tavern, Strand, on Wednesday, July 6th, 1825, at three o'clock in the afternoon.—The chair to be taken at Four o'clock precisely

The Anniversary Dinner will be on the same day at the above Tavern, at six o'clock. Stewards;

| | |
|---|---|
| Henry Blatch, Esq. | Rich. H. King, Esq. |
| Geo. Fincham, Esq. | Jas. Morrah, Esq. |
| Wm. Hilman, Esq. | Jos. Rose, Esq. |
| Thos. Hurst, Esq. | Geo. Woolley, Esq. |

Those members or their friends who intend favouring the Stewards with their company, are requested to signify their intention by letters directed to Mr. Ottey, Crown and Anchor Tavern, post paid, on or before Tuesday, July 5, 1825.

Ticket 20*s*. ——— J. POWELL, Secretary.

Dr. Barry, an English physician and army surgeon, has lately read a Memoir on the Circulation of the Blood, before the Royal Academy of Medicine, at Paris. From a great number of experiments on animals, and especially on horses, he has come to the conclusion that the return of blood by the veins is caused by direct atmospheric pressure. Our limits prevent us at present from doing more than announce the subject of the memoir.

---

*Penetrating Wounds of the Chest.* [Revue Med. Mars, 1825.] M. Toulmouche has published two cases of this kind, with some reflections, which we deem worthy of notice.

*Case* 1. In the month of August, 1823, Renault, a carpenter, aged 26 years, fell out of a bed on which he was lying, and the point of a file in his waistcoat-pocket entered his chest and penetrated a considerable way. By the fall the file broke at its junction with the handle. Some hæmorrhage ensued, and continued for two hours after the patient had the courage to extract the file from the wound. His respiration was a good deal embarrassed at the time. A surgeon was called, and applied a cupping glass to the orifice of the wound. During the first month a sanguineo-serous discharge took place from the wound, and the patient was easy in proportion to the quantity of discharge. Gradually the nature of the discharge altered, and became purulent. During the two or three succeeding months there was constant fever, and the patient could not lie on the wounded side, without danger of suffocation. In this situation, Renault was admitted into the hospital at Rennes. The chest had not hitherto been examined by means of auscultation or percussion, and the only thing done at first, in the hospital, was to dilate the wound, from which there issued an abundance of sero-purulent matter, with but temporary advantage. Emaciation slowly advanced; and tired out, he left the hospital and returned to his native country. It was on the 8th of December, 1824, that our author was invited to see this poor wretch, at a small village in the country. He was then pale and emaciated—debility extreme—respiration very difficult, and only to be carried on in the perpendicular posture—copious expectoration of purulent matter from an opening between two of the ribs on the left side of the chest. A probe was introduced its whole length, but failed to reach the bottom. Our author had no stethoscope with him, and could not examine the chest properly till five days afterwards. *Examination.* Excepting at the anterior and superior part of the left side of the chest, the sound, on percussion, was dull as marble. The right side sounded clear. The left side of the thorax was bulged out a little, and the intercostal spaces were broader than on the right side. By auscultation it was observed that the respiratory murmur was only audible in the part that sounded well, that is, under the clavicle about one third way down the sternum. Succussion produced on the ear a faint sound of fluctuation. There was nothing particular in the action of the heart. The following diagnosis was pronounced:—*Adhesion of the left lung to the parietes of the chest at the upper part—abundant sero-purulent effusion with pneumothorax in the other part of the left side.* The patient was transported to the hospital, where he almost instantly died. The dissection verified the diagnostic. When a scalpel was plunged in between two of the ribs on the left side, there escaped a quantity of gas. When the sternum was removed, the left lung was found squeezed up and adherent at the superior portion of the chest by false membranes of long standing. False membranes also lined the parietes of this side below, where three pints, and more, of a yellowish pale sero-purulent fluid were collected. The wound communicated with this cavity. The other appearances were of minor importance, and need not here be enumerated. M. Toulmouche's reflexions are not particularly interesting.

*Case* 2. M. Adolphe K——— 22 years of age, a law-student, received, on the 4th July, 1823, in a duel, a thrust of a small sword, half an inch below the right clavicle. He fell to the ground vomiting florid blood. He was transported to a cottage and medical aid procured. There was but little hæmorrhage from the wound. He was bled before our author arrived. The track of the wound could not be ascertained. There was some slight emphysema—the sense of suffocation was great—each paroxysm of coughing was accompanied by the discharge of a quantity of frothy blood—the countenance was pale—the lips without colour—extremities cold—pulse quick. The vein was again opened, and some more blood abstracted. The discharge of blood ceased, but the breathing continued difficult—and some delirium was present. In the evening the patient was more quiet, and could lie on either side—the chest sounded equally well on both sides. Leeches were applied round the wound. The night was calm, but the breathing was occasionally stertorous. *5th July*. Same state—the patient was plunged in a soporose condition, and would hardly answer questions, except by pointing to his wound. His intellects now became completely obscured. A consultation of physicians was called. The breathing was very difficult, and accompanied by a rattling in the throat. The cutaneous emphysema was now considerable. Died at 7 o'clock in the evening.

*Dissection*. The sword had penetrated the summit of the right lung, passing near the arteria innominata—pierced the trachea between the ninth and tenth cartilaginous rings, and issued between the 12th and 13th, wounding the œsophagus, but not penetrating into its cavity. There were effusion and infiltration of blood in the muscular parietes near the wound. The right lung adhered to the side by old membranes. The parenchymatous structure of the lung was gorged—and some blood effused under the pleural covering. The other side of the chest was sound.

### MR. WEISS's INSTRUMENTS.*

Having a short time ago undertaken to lay before the public a new Syringe for extracting poison from the stomach, and for administering enemas in any quantity, I have the satisfaction to announce that I have invented an instrument of this description, which will be found superior to any heretofore produced. It is so contrived as to act without VALVES, which, as every one knows, are liable, not only to get out of order, but to be clogged up, when any particle of food is drawn from the stomach, or when any glutinous substance is injected. My Syringe, on the contrary having merely a common cock, is liable to none of those disadvantages, and is so simple in its construction, that the operator, by merely turning the handle, may fill or empty it through whichever aperture he pleases, without the inconvenience imposed by valve syringes, of changing the tubes, which must necessarily distract his attention.

The annexed is a representation of a new Lever and Forceps which I invented about two years ago, and which was pronounced to be very excellent by a certain practitioner of midwifery, who, however, recommended the construction of a pair of forceps on the same principle, which might be used instead of long forceps; I accordingly constructed a pair for him, to his entire satisfaction, but of which he now claims the invention as his own, unjustly as I think, since I may surely consider myself entitled to the benefit and credit of any instrument which I have invented, and on which I have expended my time and my property. This instrument is so simple as to require very little explanation:—By merely lifting the button C from the notch, the operator may give to it whatever angle he pleases. The lever may be either curved or straightened, by turning or reverting the screw at the bottom of the handle. They may both be introduced in a straight direction.

* See Plates with this Number.

## Figures and Tables

**Figure f1:**
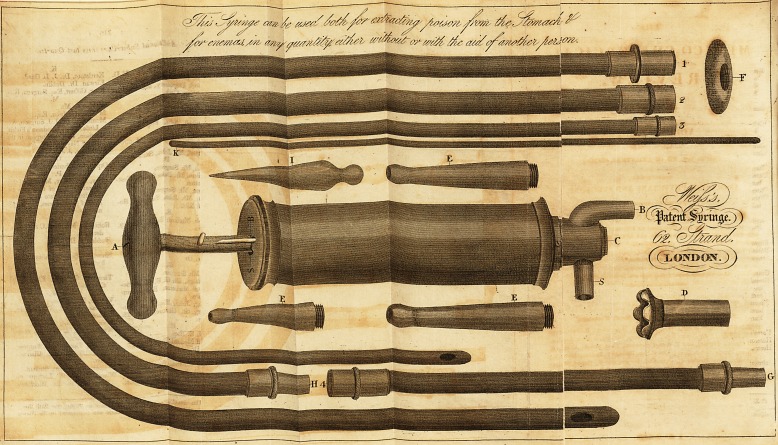


**Figure f2:**